# Impaired Mitochondrial Energy Metabolism Regulated by *p70S6K*: A Putative Pathological Feature in Alzheimer’s Disease

**DOI:** 10.3390/metabo14070369

**Published:** 2024-06-29

**Authors:** Wenyu Gu, Xinli Cong, Yechun Pei, Nuela Manka’a Che Ajuyo, Yi Min, Dayong Wang

**Affiliations:** 1 Key Laboratory of Tropical Bioresources of the Educational Ministry of China, School of Pharmaceutical Sciences, Hainan University, Haikou 570228, China; 2 Laboratory of Biopharmaceuticals and Molecular Pharmacology, One Health Cooperative Innovation Center, Hainan University, Haikou 570228, China; 3 Department of Biotechnology, School of Life and Health Sciences, Hainan University, Haikou 570228, China

**Keywords:** Alzheimer’s disease, p70S6K, oxidative phosphorylation, secreted Aβ42, RNA sequencing, mitochondrial energy metabolism

## Abstract

Alzheimer’s disease (AD) is a neurodegenerative disease. Mitochondrial energy metabolism and p70 ribosomal protein S6 kinase (p70S6K) play significant roles in AD pathology. However, the potential relationship between them is unclear. In this study, bioinformatics methods were initially applied to analyze the transcriptomic data in the CA1 and the primary visual cortex of patients with AD and Aβ42-treated SH-SY5Y cells. By applying secreted Aβ42 and *p70S6K* gene silencing in cells, we explored disorders in mitochondrial function and the regulatory roles of p70S6K by flow cytometry, laser scanning confocal microscopy, high-performance liquid chromatography, Western blotting, and quantitative reverse transcription PCR. The study reveals that impaired mitochondrial energy metabolism is a potential pathological feature of AD and that *p70S6K* gene silencing reversed most of the changes induced by Aβ42, such as the activities of the electron transport chain complexes I and III, as well as ATP synthase, ATP production, generation of reactive oxygen species, mitochondrial membrane potential, and phosphorylation of AMPK, PINK1, and Parkin, all of which are required for mitochondria to function properly in the cell.

## 1. Introduction

Alzheimer’s disease (AD) is a neurodegenerative disease characterized by the deposition of amyloid–β (Aβ) in the brain. Aβ1–42 (Aβ42) is the primary component that plays an essential role in the pathogenesis of AD, and it is believed to be the main cause of AD [1]. AD pathology includes substantial neuronal loss [2], synaptic degeneration [3], DNA damage [4], metabolic dysfunctions [5], neuroinflammation [6], mitochondrial dysfunction [7], and oxidative stress [5]. However, the pathogenesis of AD is complicated, and current research suggests that severe metabolic dysfunction may be a pathognomonic marker of AD [8]. The mitochondrial hypothesis is a potential mechanism underlying the pathogenesis of AD [9]. The relationship between mitochondrial dysfunction and AD can be explained by abnormalities in mitochondrial energy metabolism, biogenesis, axonal transport, fusion, and fission processes, as well as autophagy [10,11]. Impaired mitochondrial function contributes to AD progression because the brain is the largest consumer of ATP in the body, accounting for more than 20% of the total energy available [12,13,14]. Electron transport chain (ETC) malfunctions result in increased ROS generation; oxidative stress is responsible for causing damage to neurons and is believed to be the hub linking the various pathogeneses of AD [15,16]. All of these factors are intricately correlated with AD pathology. It has also been found that damaged mitochondria accumulate in APP/PS1 mice and that increased mitochondrial autophagy reduces Aβ deposition and ameliorates cognitive dysfunction in APP/PS1 mice [17]. Thus, mitochondrial energy metabolism and mitochondrial autophagy can be targets for the treatment of AD.

p70 Ribosomal protein S6 kinase (p70S6K) is a serine/threonine kinase that responds to mammalian target of rapamycin (mTOR) signaling to promote protein synthesis, cell growth, and cell proliferation [18]. The inhibition of p70S6K can attenuate BACE1 protein synthesis and enhance synaptic plasticity [19]. p70S6K is also an insulin signaling effector, the feedback of which inhibits insulin–receptor substrate 1 (IRS–1) and attenuates the effects of insulin, and dysregulated insulin signaling is a key feature of AD [20]. Our previous study found that phosphorylation levels of p70S6K Thr389 were significantly increased in the brains of aged APP/PS1 mice. From this, it can be seen that p70S6K may be an important therapeutic target for AD. The pathogenic roles of p70S6K in AD remain unclear. The study aims to better understand mitochondrial dysfunction in AD and the regulatory role of p70S6K in it.

## 2. Materials and Methods

### 2.1. Cell Culture 

Neuroblastoma SH–SY5Y cells and embryonic kidney HEK 293 cells were obtained from the China Center for Type Culture Collection, and they were cultured in Dulbecco’s Modified Eagle Medium (DMEM) (Thermo Fisher Scientific, Suzhou, China) containing 10% FBS (Biological Industries, Beit HaEmek, Israel) and 1% penicillin/streptomycin (Thermo Fisher Scientific, Shanghai, China). Cells were grown to 80%–90% confluence and then treated with drugs.

### 2.2. Plasmid Transfection and Cell Treatments

The Aβ42–pcDNA3.1(+) plasmid, which has the secretion signal peptide sequence, was transfected into HEK293 cells to express Aβ42 only. The pcDNA3.1(+) blank plasmid served as the control. The plasmids and PEI (Servicebio, Wuhan, China) working solution were prepared in serum–free medium, mixed well, and then added to HEK293 cells after 15 min of incubation at room temperature. After 6 h, the medium was replaced with complete medium for 48 h. For siRNA transfection, serum–free medium was used to prepare siRNA working solution and Lipo6000 (Beyotime Biotechnology, Shanghai, China) working solution separately, which were incubated for 5 min, mixed and incubated for another 5 min, and then added to the SH–SY5Y cells. Six hours later, the cells were replaced with complete medium and cultured for 48 h. HPLC–purified double–strand siRNA targeting the sequence ‘AAAGAGAGGGAATATTTA’ of the *p70S6K* gene was used in the study; the sense sequence of the *p70S6K* siRNA is ‘GAAAGAGAGAGGGAAUAUUUUATT’, and the antisense sequence is ‘UAAAUAUUUCCCUCUCUUUUCTT’. The cells were first transfected with siRNA to knock down the expression of *p70S6K*, and Aβ42 was then added to the SH–SY5Y cells. Twenty–four hours later, the cells were used in the experiments. 

### 2.3. RNA Extraction, Reverse Transcription, and RT–qPCR

The SH–SY5Y cells were seeded at a density of 1 × 10^6^ cells per well in 6–well plates. When the cell confluency reached more than 80%, the cells were transfected with *p70S6K* siRNA before being treated for 24 h with secreted Aβ42. After cell treatment, the total cellular RNA was extracted. Total RNA was extracted from the SH–SY5Y cells using Trizol kits (Sangon Biotech, Shanghai, China), and the RNA was resolved by 0.9% agarose gel electrophoresis for quality control. One microgram of RNA from each sample was sent to Majorbio for transcriptome sequencing, and the remaining RNA was reverse transcribed to cDNA for RT–qPCR using a reverse transcription kit (SparkJade, Jinan, China). The SYBR™ green RT–qPCR master mix (Beyotime Biotechnology, Shanghai, China) and the Roche LightCycler^®^ 480 system (Vienna, Austria) was used for the RT–qPCR analysis. The RT–qPCR primers are listed in Table 1.

### 2.4. Protein Extraction and Western Blot

Cells were treated as described in Section 2.3, after which the total protein was extracted. A total of 200 μL of cell lysis buffer (10 mM Tris–HCl; 100 mM NaCl; 1 mM EDTA; 1 mM EGTA; 20 mM NaF; 1 mM Na_3_VO_4_; 1% Triton X–100; 10% glycerol; 1% sodium deoxycholate; and protease and phosphatase inhibitor cocktails, pH 7.4) was added to each 10 cm^2^ for incubation on ice for 15 min. Then, it was transferred into 1.5 mL EP tubes, followed by centrifugation at 12,000 rpm for 20 min at 4 °C. The supernatant, which contains cellular proteins, was collected. The total protein concentration was determined and normalized using BCA assay kits (Takara Bio, Beijing, China). The proteins were separated using SDS–PAGE and transferred to nitrocellulose membranes by wet transfer (transfer buffer: 25 mM Tris; 192 mM glycine; 0.1% SDS; and 20% (*V*/*V*) methanol). The transfer was conducted for 90 min with the conditions set to 100 V and a maximum current of 350 mA; then, the membranes were blocked with a 5% nonfat milk solution at room temperature for 2 h, followed by incubation overnight at 4 °C with antibodies. After incubation, the membranes were washed three times with TBST solution for 10 min each and then incubated with secondary antibodies for 1.5 h at room temperature. They were visualized using a chemiluminescence imaging system after three additional washes with TBST buffer for 10 min each. The specific antibodies used in this study were anti–Parkin (1:1000, Affinity Biosciences, Liyang, China), anti–PINK1 (1:1000, Affinity Biosciences, Liyang, China), anti–SQSTM1/p62 (1:1000, Affinity Biosciences, AF5384), anti–LC3B (1:1000, Abcam, Cambridge, UK), anti–p–AMPK α1Thr183/α2Thr172 (1:2000, Beyotime Biotechnology, Shanghai, China), β–actin (1:1000, Servicebio, Wuhan, China), anti–p–p70S6K Thr389 (1:1000, Cell Signaling Technology, Shanghai, China), HRP–conjugated goat anti–rabbit IgG (1:3000, Beyotime Biotechnology, Shanghai, China), and HRP–conjugated goat anti–mouse IgG (H+L) (1:1000, Beyotime Biotechnology, Shanghai, China).

### 2.5. HPLC Determination of ATP Level

Cells were treated as described in Section 2.3, and the ATP levels were assayed after the cell treatment. A total of 10 mM ATP (Solarbio, Beijing, China) was used as the external standard, and the ATP levels of the samples were determined by HPLC. The chromatographic conditions were as follow: the mobile phase was a 0.05 M KH_2_PO_4_ buffer, pH 7; the detection wavelength was 259 nm; the flow rate was 1.0 mL/min; the column temperature was maintained at 25 ± 5 °C; and the injection volume was 20 μL. The ATP concentration was calculated by comparing the peak area of a sample to that of the standard curve.

### 2.6. Flow Cytometry Analysis of Reactive Oxygen Species (ROS)

Cells were treated as described in Section 2.3, and then a flow cytometer was used to measure the ROS levels. The fluorescent probe DCFH–DA was utilized for the detection using the CellROX green reagent kit (Beyotime Biotechnology, S0033M). After treatment, the cells were washed three times with DPBS, trypsinized, collected, and centrifuged at 1500 rpm for 5 min and then resuspended in DPBS. The cellular fluorescence intensity was quantified by flow cytometry using the FITC channel in order to characterize the cellular ROS levels. 

### 2.7. Analysis of Activities of Electron Transport Chain (ETC) Complexes

Cells were handled in the same way as described in Section 2.3. The activities of the key components of the electron transport chain complexes were detected using the following testing kits: NADH dehydrogenase E–BC–K834–M, Elabscience, Houston, TX, USA), succinate dehydrogenase (E–BC–K835–M, Elabscience, Houston, TX, USA), cytochrome c reductase (E–BC–K151–M, Elabscience, Houston, TX, USA), cytochrome C oxidase (E–BC–K152–M, Elabscience, Houston, TX, USA), ATP synthase (E–BC–K153–M, Elabscience, Houston, TX, USA), and NAD^+^/NADH (E–BC–K804–M, Elabscience, Houston, TX, USA), using the protocols provided by the manufacturer.

### 2.8. Laser Confocal Microscopic Detection of Mitochondrial Membrane Potential (MMP)

One day before the treatment, SH–SY5Y cells were seeded in 96–well plates at a density of 5 × 10^4^ cells per well. *p70S6K* siRNA was transfected into the cells for 48 h. Then, the cells were treated for 24 h with Aβ42 produced by HEK 293 cells, and JC–1 test kits (Beyotime Biotechnology, Shanghai, China) were used to determine the MMPs, which were visualized using a laser confocal microscope.

### 2.9. Bioinformatics Analysis

This study’s data were gathered through transcriptome RNA sequencing and from the GEO datasets GSE184942 and GSE203206. The RNA sequencing of the SH–SY5Y cells was conducted by Majorbio (Shanghai, China). The GO, KEGG pathway, GSEA, and DO analyses were performed using the online Majorbio Cloud Platform (access date: 1 March 2023). DESeq2 software (version 1.44) was used for the differential analysis, with a screening threshold of |log_2_FC| > 1 (FC = fold change) and *p*-values less than 0.05. The following website was used to acquire the reference genes: http://asia.ensembl.org/Homo_sapiens/Info/Index, access date: 31 December 2022.

### 2.10. Statistical Analysis

The Western blot (WB) and fluorescence results were analyzed using NIH ImageJ software (version 1.53) bundled with 64–bit Java 8. To calculate the WB results, the intensity of each target band on a sheet of nitrocellulose membrane was generalized to the sum of all target bands on the sheet to minimize the batch error. Then, the generalized data for the control group were averaged to obtain the control mean value (CMV). The generalized data for each group were compared to the CMV to obtain individual ratio values, which were used in the statistical analyses, which were performed using either the Student’s *t*–test or one–way analysis of variance (ANOVA), followed by the Tukey–Kramer post hoc test for multiple comparisons. Graphical representations were generated using Microsoft Excel or GraphPad Prism 9 software. Statistical significance was defined as a *p*-value less than 0.05.

## 3. Results

### 3.1. Aβ42 Affects Diverse Pathways in SH–SY5Y Cells and Is Implicated in Multiple Diseases

**Figure 1:**
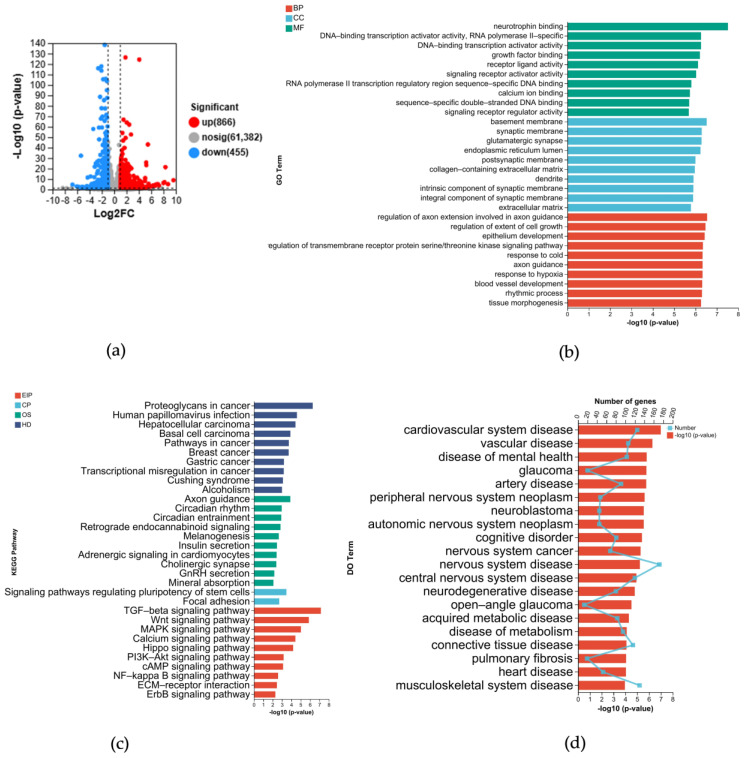
DEG enrichment in the Aβ42–treated group versus the control group. (**a**) Volcano map of DEGs in the Aβ42–treated group. The red dots represent upregulated gene expressions; blue dots indicate downregulated gene expressions; and gray dots represent nonsignificant gene expressions. (**b**) Top DEG clusters in the BP, CC, and MF aspects of the Gene Ontology. (**c**) Top DEG clusters in the EIP, OS, HD, and CP aspects of the KEGG pathway. (**d**) Top 20 DEG clusters in the Disease Ontology (DO). The dots on the folded line represent the numbers of genes in the respective DO terms.

As the amyloid cascade hypothesis is widely recognized and Aβ42 is considered a key factor in AD, this study first investigated the effect of Aβ42 on the biological processes of SH–SY5Y cells. In this study, RNA sequencing of SH–SY5Y cells was conducted by Majorbio, and the resulting data were subsequently analyzed using the online Majorbio Cloud Platform. The differentially expressed genes (DEGs) between the Aβ42–treated group and the control were first screened for |log_2_FC| ≥ 1, *p* < 0.05, and volcano plots of the DEGs were drawn (Figure 1a). The results show that 866 genes were upregulated and 455 genes were downregulated in expression. The top 10 enriched GO terms in the domains of biological process (BP), cell component (CC), and molecular function (MF) are shown in Figure 1b. The terms in BP are mainly concerned with cell growth, development, and regulation of axons. The most clustered term in CC is synapse. The MF indicates the transduction of signaling molecules and the transcriptional activity. The Kyoto Encyclopedia of Genes and Genomes (KEGG) pathway enrichment revealed the top 10 terms in the categories of environmental information processing (EIP), organismal systems (OS), human diseases (HD), and cellular process (CP) (Figure 1c). The results also show that Aβ42 induces upregulation in multiple signaling pathways, such as TGF–beta, Wnt, and MAPK in SH–SY5Y cells. In addition, they indicate that multiple pathways may also be involved in cancer. The top 20 Disease Ontology (DO) terms included cardiovascular disorders, neurological disorders, neoplasms, metabolic disorders, and others (Figure 1d). Aβ42 had the most significant effect on the nervous system, followed by cardiovascular disease. Most of the above diseases have been reported to be associated with AD. The above results suggest that Aβ42 can cause changes in multiple pathways in SH–SY5Y cells, and these pathways may be involved in multiple diseases; therefore, the risk factors and pathologies in AD are complex, and elucidating the pathological mechanisms of AD may also provide therapeutic tools for other diseases.

### 3.2. Impaired Mitochondrial Energy Metabolism in AD Revealed by Transcriptome Analysis

AD is caused by multiple factors and leads to multiple physiologies, and the effect of Aβ42 on SH–SY5Y cells does not fully explain the pathological mechanisms of AD. This study further used transcriptomic data from the GSE184942 and GSE203206 datasets in the GEO database and analyzed them together with the transcriptomic data from SH–SY5Y cells to more accurately explore the key physiology of AD. GSE184942 contains transcriptomic data from the CA1 region of the hippocampus of patients with AD and healthy individuals. GSE203206 contains transcriptomic data from the visual cortex of patients with AD and healthy individuals. Atypical regions, such as the visual cortex, and atypical clinical symptoms, such as visual disturbances, can also reflect transcriptomic features of traditionally pathologized regions. This provides a unique opportunity to understand AD transcriptomic features [21].

**Figure 2:**
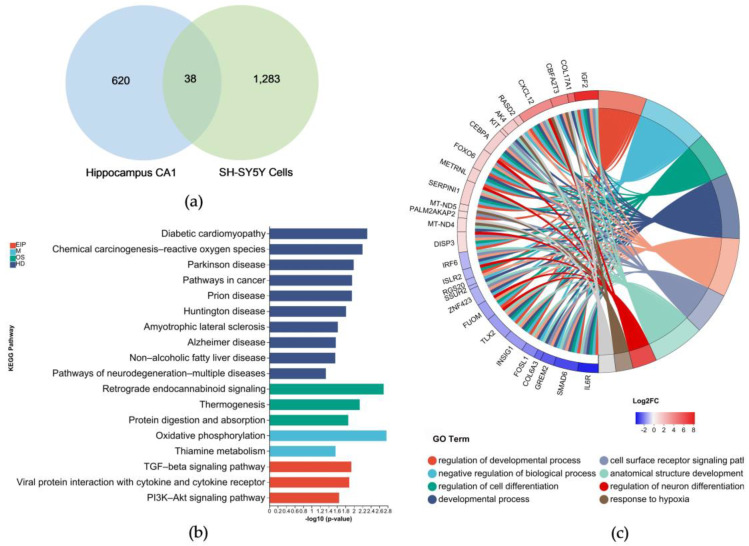
GO and KEGG pathway analyses of common DEGs in the hippocampal CA1 area in patients with Alzheimer’s disease and Aβ42–treated SH–SY5Y cells: (**a**) Venn diagram showing the DEGs in the hippocampal CA1 area of patients with Alzheimer’s disease and Aβ42–treated SH–SY5Y cells; (**b**) top clusters of common DEGs in the EIP, OS, HD, and M sections of the KEGG pathway; (**c**) chord plots showing the top clustered GO terms for the common DEGs, and the genes.

A Venn diagram shows the 38 common DEGs shared by the cells and the patients’ CA1s (Figure 2a). The chord plot displaying the GO enrichment of the 38 genes enables the visualization of genes with a significant contribution. The top 10 terms are classified into EIP, OS, HD, CP, and metabolism (M) KEGG pathways (Figure 2b). The KEGG pathway clusters are related to the following two major subjects: one is neurodegenerative diseases, such as Parkinson’s, prion diseases, retrograde endogenous cannabinoid signaling, and AD; the other is related to mitochondria, including oxidative phosphorylation, ROS, and heat production. The top 10 GO terms are primarily related to cell growth and development and oxygen levels (Figure 2c). The results of the GO and KEGG analyses indicate that the major pathologies in AD are related to mitochondrial function, especially from an OXPHOS aspect.

**Figure 3:**
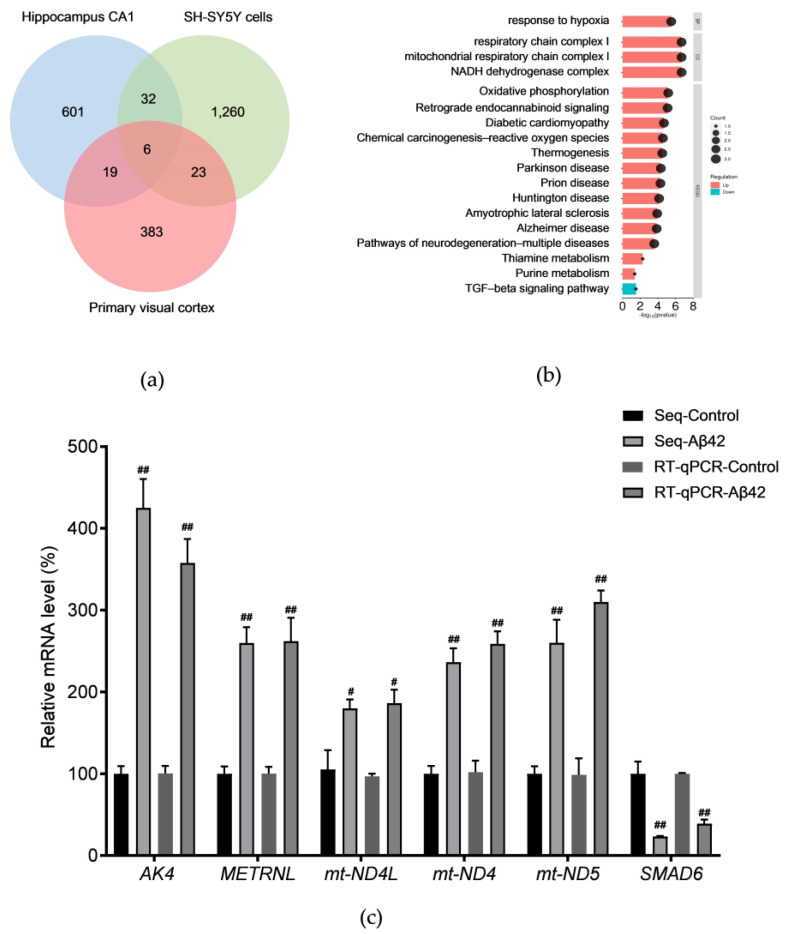
GO and KEGG pathway analyses of the common DEGs in the hippocampal CA1 area and primary visual cortex of patients with Alzheimer’s disease and Aβ42–treated SH–SY5Y cells: (**a**) Venn diagram showing the DEGs in the hippocampal CA1 area and primary visual cortex of patients with Alzheimer’s disease and Aβ42–treated SH–SY5Y cells; (**b**) top clusters of common DEGs in the KEGG pathway, where the pink color signifies upregulated gene expressions; blue represents downregulated gene expressions; and black dots signify the numbers of genes enriched in respective terms; (**c**) transcriptome sequencing and qRT–PCR analyses of the transcription levels of *AK4*, *mt–ND4*, *mt–ND4L*, *mt–ND5*, *SMAD6*, and *METRNL* genes. ^#^ *p* < 0.05 and ^##^ *p* < 0. 01. compared with the respective control group by one–way ANOVA, followed by the Tukey–Kramer post hoc test for multiple comparisons. The results from three independent experiments are expressed as the means ± SD.

Next, the key genes in AD were further investigated by incorporating transcriptomic data from the primary visual cortex. The DEGs for the SH–SY5Y cells, CA1, and primary visual cortex are plotted in a Venn diagram (Figure 3a), and six DEGs are common to all three, namely, *AK4*, *METRNL*, *mt–ND4L*, *mt–ND4*, *mt–ND5*, and *SMAD6*, respectively. Among them, *mt–ND4*, *mt–ND4L*, and *mt–ND5* are mitochondria–encoded DNAs (mt–DNA). The results of the GO and KEGG analyses of the six genes indicate a connection to oxidative phosphorylation, ETC, and several neurodegenerative diseases, including AD, Huntington’s disease, and Parkinson’s disease (Figure 3b). The mRNA expression levels for *AK4*, *METRNL*, *mt–ND4L*, *mt–ND4*, *mt–ND5*, and *SMAD6* were verified using RT–qPCR, and the RT–qPCR results are consistent with the transcriptome sequencing results. *SMAD6* was downregulated, while *AK4*, *METRNL*, *mt–ND4L*, *mt–ND4*, and *mt–ND5* showed upregulation (Figure 3c). AK4 is an adenylate kinase found in the mitochondrial matrix. It is responsible for maintaining the balance of cellular nucleotides and has a protective function in the cellular response to oxidative stress [22,23]; *METRNL* promotes energy expenditure, thermogenesis, and improves glucose homeostasis [24]; *mt–ND4L*, *mt–ND4*, and *mt–ND5* encode different NADH–ubiquinone oxidoreductase chains in an ETC. Upregulation of the expressions of *AK4, METRNL*, *mt–ND4L*, *mt–ND4*, and *mt–ND5* and downregulation of the expression of *SMAD6* suggest abnormalities in mitochondrial function. Increases in the mt–DNAs’ copy numbers at an early stage of the disease could be a compensatory mechanism [25]. These findings imply that energy metabolism disorders occur in AD and that mitochondria may be an essential target for AD treatment. 

### 3.3. Influence of Aβ42 and p70S6K on OXPHOS in SH–SY5Y Cells

**Figure 4:**
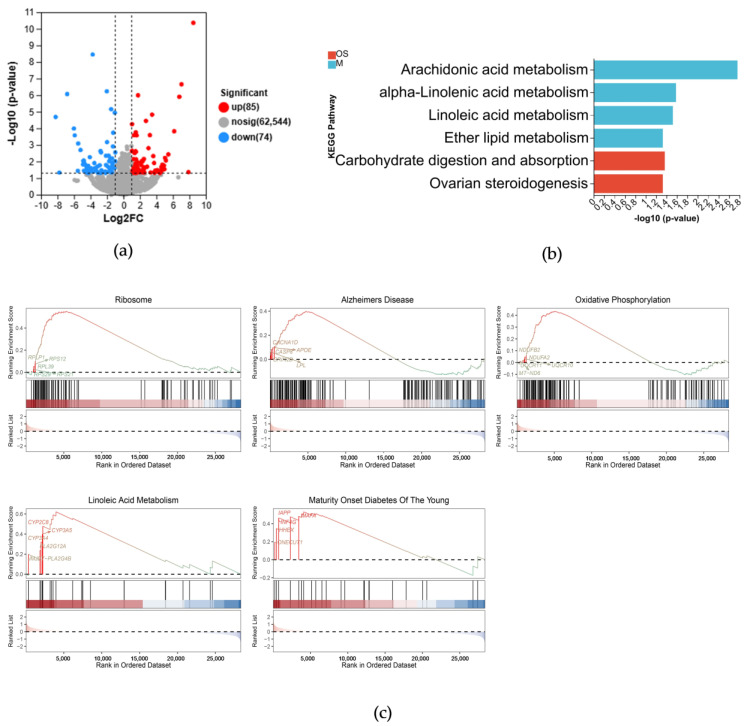
Differential gene expressions induced by *p70S6K* gene knockdown in Aβ42–treated cells: (**a**) volcano map of the DEGs in the *p70S6K* siRNA and the Aβ42–treated group, and the Aβ42–treated control group, where red dots represent upregulated gene expressions; blue dots indicate downregulated gene expressions; and gray dots show nonsignificant gene expressions; (**b**) KEGG pathway analysis of the DEGs; (**c**) top 5 enriched terms of the GSEA of DEGs.

The role of p70S6K in AD was further analyzed by bioinformatics. Aβ42+p70S6K siRNA had a total of 159 DEGs compared to the Aβ42 group (Figure 4a). The results of the KEGG analysis (Figure 4b) were mainly enriched in lipid metabolism. The top five pathways were ribosome, linoleic acid metabolism, oxidative phosphorylation, AD, and maturity onset diabetes of the young (Figure 4c). Notably, p70S6K is associated not only with AD but also with oxidative phosphorylation. The analysis showed that the genes associated with OXPHOS are *NDUFA2*, *NDUFB2*, *UQCR10*, *UQCR11*, and *mt–ND6*. *NDUFA2* and *NDUFB2* encode subunits of NADH dehydrogenase, while *UQCR10* and *UQCR11* encode subunits of cytochrome c reductase, and *mt–ND6* encodes NADH–ubiquinone oxidoreductase chain 6. Together with the results presented in Section 3.2, it is indicated that p70S6K is intricately involved in mitochondrial energy metabolism in AD.

### 3.4. Influence of Aβ42 and p70S6K on Levels of NAD^+^, NADH, ATP, and ROS

**Figure 5:**
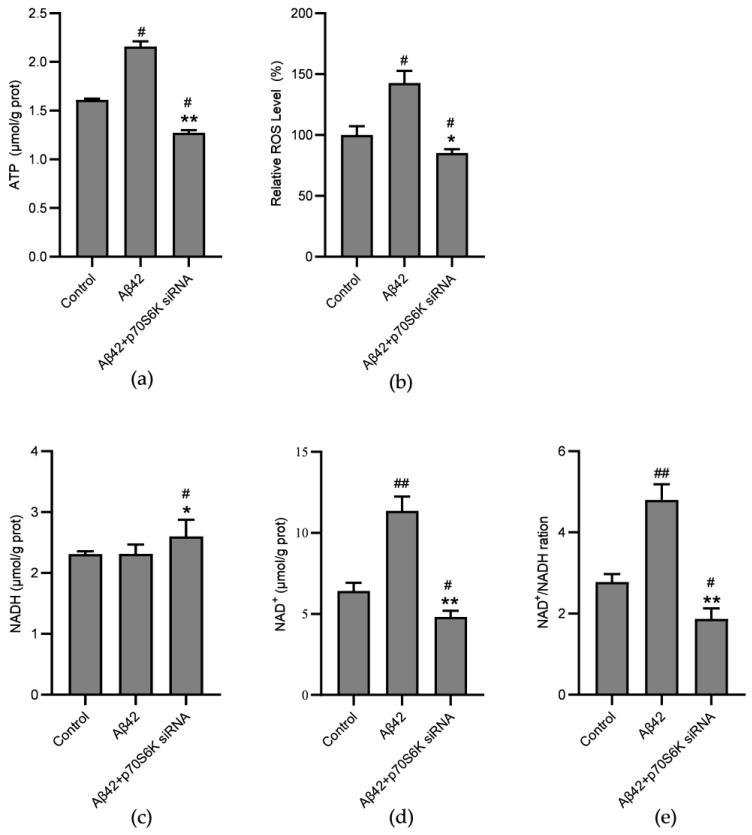
Effects of the *p70S6K* gene knockdown on the levels of NAD^+^, NADH, ATP, and ROS in Aβ42–treated SH–SY5Y cells: (**a**) HPLC assay of the ATP levels in *p70S6K* siRNA and the Aβ42–treated SH–SY5Y cells; (**b**) ROS levels detected by flow cytometry in the cells; (**c**) levels of NADH in the cells; (**d**) levels of NAD^+^ in the cells; (**e**) ratio of NAD^+^/NADH in the cells. The SH–SY5Y cells were treated with Aβ42 for 24 h with or without knockdown of the *p70S6K* gene expression. * *p* < 0.05 and ** *p* < 0.01, compared with the Aβ42–treated group; ^#^ *p* < 0.05 and ^##^ *p* < 0.01 compared with the control group, by one–way ANOVA, followed by the Tukey–Kramer post hoc test for multiple comparisons. The results from three independent experiments are expressed as the means ± SD.

NAD^+^ accepts hydrides produced by heterolytic bond cleavage to produce NADH, which transports protons and electrons to the NADH oxidoreductase, the main component of ETC complex I. ATP is the most significant metabolite produced in mitochondria. We analyzed the effects of *p70S6K* gene silencing on intracellular levels of NAD^+^, NADH, ATP, and ROS, which were found to be dysregulated in AD. The results demonstrate that treating the cells with Aβ42 for 24 h increased the levels of both ATP (Figure 5a) and ROS (Figure 5b). Graphs of the HPLC and the flow cytometry assay results can be found in the Supplementary Materials. The levels of NADH remained unchanged (Figure 5c), while the levels of NAD^+^ increased (Figure 5d), resulting in an elevated NAD^+^/NADH ratio (Figure 5e). The results indicate that Aβ42 induces oxidative stress. However, after a short period of stimulation, the ATP levels and the NAD^+^/NADH ratio increased, indicating that the cell might initiate rescue mechanisms at the time. Within the cells, silencing of the *p70S6K* gene inhibited the oxidative stress response and reversed the increases in ATP levels and NAD^+^/NADH ratios induced by Aβ42. 

### 3.5. Influence of Aβ42 and p70S6K on Activities of Mitochondrial ETC Complexes

**Figure 6:**
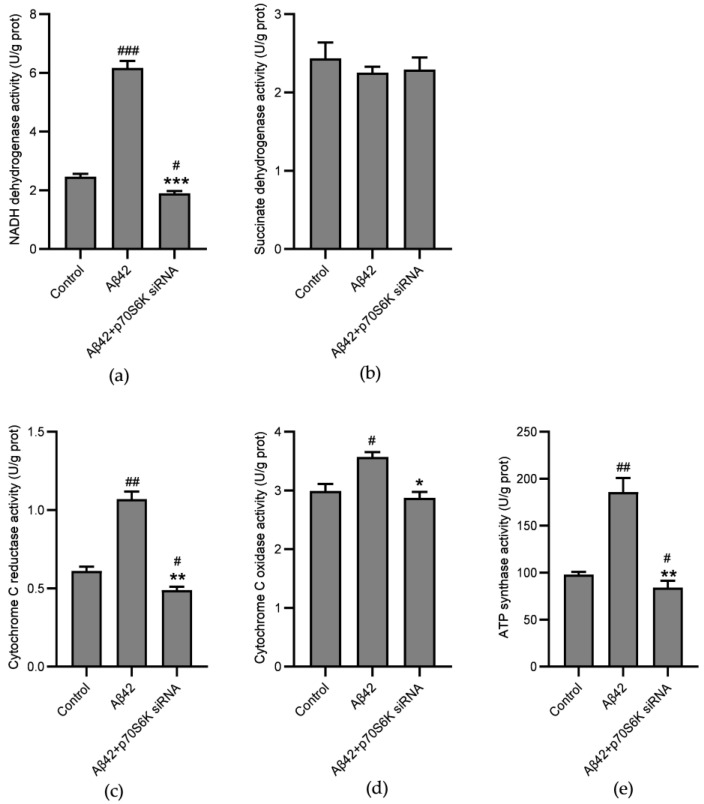
Effects of the *p70S6K* gene knockdown on the activities of the ETC complexes in Aβ42–treated SH–SY5Y cells: (**a**) NADH dehydrogenase activities in the *p70S6K* siRNA and the Aβ42–treated SH–SY5Y cells; (**b**) succinate dehydrogenase activity in the cells; (**c**) cytochrome c reductase activities in the cells; (**d**) cytochrome C oxidase activities in the cells; (**e**) ATP synthase activities in the cells. The SH–SY5Y cells were treated with Aβ42 for 24 h with or without knockdown of the *p70S6K* gene expression. * *p* < 0.05, ** *p* < 0.01 and *** *p* < 0.001, compared with the Aβ42–treated group; ^#^ *p* < 0.05, ^##^ *p* < 0.01 and ^###^ *p* < 0.01, compared with the control group, by one–way ANOVA, followed by the Tukey–Kramer post hoc test for multiple comparisons. The results from three independent experiments are expressed as the means ± SD.

The elevations in the ratio of NAD^+^/NADH and the ATP level clearly suggest that the electron transport activities exhibited by the ETC complexes and the efficiency of OXPHOS were enhanced. The GESA analysis demonstrates that p70S6K may be linked with NADH dehydrogenase and cytochrome C reductase. On the basis of the above, we hypothesized that p70S6K may be involved in AD energy metabolism by regulating ETC complexes. The current experimental results demonstrate that the activities exhibited by NADH dehydrogenase (Figure 6a), cytochrome C reductase (Figure 6c), cytochrome C oxidase (Figure 6d), and ATP synthase (Figure 6e) increased after 24 h of treatment with Aβ42. However, the treatment did not significantly increase the activity of succinate dehydrogenase (Figure 6b), which does not accept electrons from NADH. However, the silencing of the *p70S6K* gene reversed the hyperactivation of the ETC complexes.

### 3.6. Influence of Aβ42 and p70S6K on Mitochondrial Membrane Potential (MMP)

**Figure 7:**
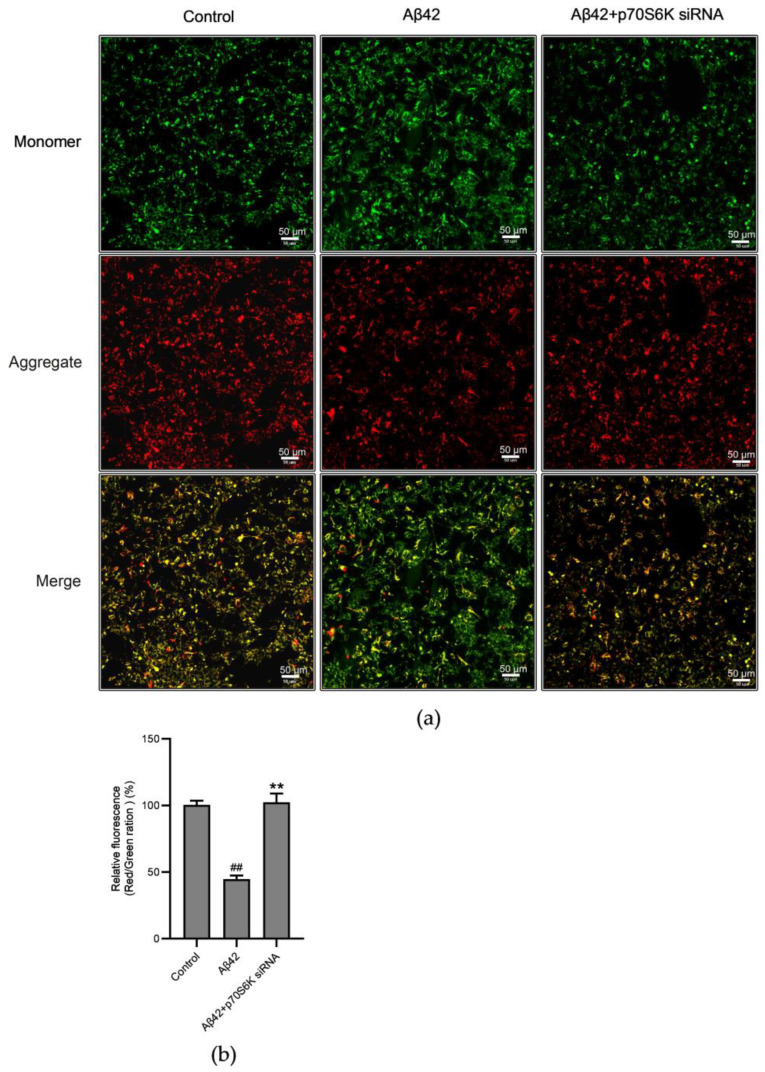
Laser confocal microscopic detection of the effects of the *p70S6K* gene knockdown on the mitochondrial membrane potential (MMP) in Aβ42–treated SH–SY5Y cells: (**a**) representative figures of the MMP detected by the JC–1 fluorescent staining; (**b**) statistical analysis of the JC–1 fluorescence intensity. The SH–SY5Y cells were treated with Aβ42 for 24 h with or without knockdown of the *p70S6K* gene expression. ** *p* < 0.01, compared with the Aβ42–treated group; ^##^ *p* < 0.01, compared with the control, by one–way ANOVA, followed by the Tukey–Kramer post hoc test for multiple comparisons. The results from three independent experiments are expressed as the means ± SD, n = 3.

The MMP reflects the electrochemical gradient of protons, which is built up by ETC complexes I, II, and IV, and it relies on the integrity of mitochondrial intermembrane. The experiment revealed an enormous decline in the MMP after 24 h of treatment with Aβ42, and the knockdown of the *p70S6K* gene reversed the decrease in the MMP induced by Aβ42 (Figure 7a,b). Along with the results demonstrating the impacts of Aβ42 on the activities of ETC complexes, these findings suggest that Aβ42 may compromise the integrity of the MMP, while knockdown of the *p70S6K* gene expression aids in maintaining the membrane integrity. 

### 3.7. Influence of Aβ42 and p70S6K on Autophagy

**Figure 8:**
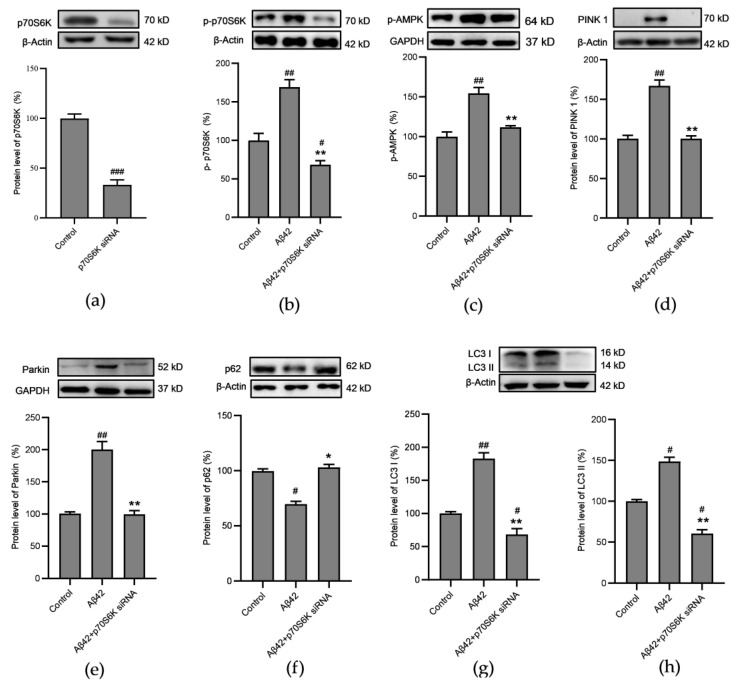
Effects of the *p70S6K* gene knockdown on the autophagy protein expression levels in the Aβ42–treated SH–SY5Y cells: (**a**) protein expression levels of p70S6K in *p70S6K* siRNA and the Aβ42–treated SH–SY5Y cells; (**b**) p70S6K Thr389 phosphorylation levels in the cells; (**c**) AMPK α1 Thr183/α2 Thr172 phosphorylation levels in the cells; (**d**) protein expression levels of PINK 1 in the cells; (**e**) protein expression levels of Parkin in the cells; (**f**) protein expression levels of p62 in the cells; (**g**) protein expression levels of LC3 I in the cells; (**h**) protein expression levels of LC3 II in the cells. The SH–SY5Y cells were treated with Aβ42 for 24 h with or without knockdown of the *p70S6K* expression. * *p* < 0.05 and ** *p* < 0.01 compared with the Aβ42–treated group; ^#^ *p* < 0.05 ^##^ *p* < 0.01 and ^###^ *p* < 0.001 compared with the control, by (**a**) Student’s *t*–test or (**b**–**h**) one–way ANOVA, followed by the Tukey–Kramer post hoc test for multiple comparisons. The results from three independent experiments are shown as the means ± SD.

A dynamic equilibrium exists between mitochondrial generation and clearance, which is disrupted when cells are exposed to adverse conditions, leading to a variety of disorders. The *p70S6K* siRNA used in this study was able to effectively knock down the expression of the p70S6K protein (Figure 8a) and decrease the phosphorylation level of the p70S6K Thr389 (Figure 8b). The present results show that in the SH–SY5Y cells treated with Aβ for 24 h, the phosphorylation levels of the autophagy–related protein, AMPK, increased (Figure 8c), and the PINK 1/parkin/p62 and LC3 pathways were also activated (Figure 8d–g), indicating an enhanced capacity for autophagy by mitochondria, while knockdown of the *p70S6K* gene expression was able to reverse the enhanced autophagy triggered by Aβ42 in the cells. 

## 4. Discussion

Currently, hypotheses of AD, such as the amyloid cascade hypothesis, mitochondrial dysfunction hypothesis, and the oxidative stress hypothesis, are all based, to some extent, on the role of Aβ [26]. In the study, the effects of Aβ42 on SH–SY5Y cells were first investigated by transcriptomic analysis. The results indicate an interrelationship between cardiovascular disorders, metabolic disorders, and cancer mentioned with AD. It has been found that hypertension shows positive correlations with Aβ42 and Tau [27], and there are negative correlations between cancer and AD [28]. Insulin resistance and impaired glucose metabolism present in diabetes that are also seen in AD [29]. These findings show that Aβ42 may be relevant to a number of disorders in addition to its major role in AD, and the understanding of the pathogenesis of AD may also have implications for the treatment of other diseases. Furthermore, our findings reveal that impaired mitochondrial energy metabolism occurs in AD and that there is a significant connection between p70S6K and mitochondria, based on transcriptomic data from SH–SY5Y cells, human hippocampal CA1, and primary visual cortex areas. 

The results of our study show that *p70S6K* gene silencing reversed the Aβ42–induced increase in the levels of important metabolites involved in energy metabolism, including ATP, ROS, NAD^+^, and NADH. The brain consumes a great amount of ATP, and the severity of AD is closely related to the degree of impaired cerebral metabolism [30]. As a coenzyme, NAD^+^/NADH plays a significant role in energy metabolism and also contributes to anti–apoptotic activity, as well as antioxidant and synaptic plasticity [31]. Although ROS at a physiological concentration have protective effects on neurons [32], mitochondrial malfunction results in the overproduction of ROS, which exacerbates mitochondrial dysfunction, and they are toxic to neurons [33]. In AD, higher levels of ROS are also linked to Tau hyperphosphorylation [34], senescence [35], an inflammatory response [36], metal ion homeostasis disruption [37], and Aβ reaction cascades [38]. *p70S6K* gene silencing also reversed the hyperactivation of NADH dehydrogenase, cytochrome C reductase, cytochrome C oxidase, and ATP synthase. The activities of ETC complexes have also been reported to be abnormal in patients with AD [39]. In addition, *p70S6K* gene silencing attenuated the mitochondrial autophagy pathway activated by Aβ42 and maintained membrane integrity, which was indicated by its effects on the MMP. In response to transient stimuli, Aβ42 and oxidative stress induce the activation of the mitochondrial autophagy pathway, which helps to maintain mitochondrial homeostasis. However, sustained stimuli lead to mitochondrial damage and a subsequent decrease in mitochondrial autophagy [40]. In addition, AMPK can regulate the autophagy pathway to enhance the clearance of Aβ [41]. Although p70S6K has been studied primarily as a regulator of protein synthesis, ATP is essential in promoting protein synthesis and cell proliferation, and, thus, mitochondrial energy production, protein synthesis, and cell proliferation are mutually regulated processes [42]. Therefore, the modulation of mitochondrial energy metabolism by p70S6K has an apparent pathophysiological role in AD. 

## 5. Conclusions

Mitochondrial energy metabolism is impaired in patients with AD. In Aβ42–treated SH–SY5Y cells, it was manifested as increased oxidative stress, decreased mitochondrial membrane potential, increased activities of the ETC complexes, and activation of mitochondria autophagy. Silencing *p70S6K* gene expression can reverse these changes, suggesting that it is a potential target for AD treatment. 

## Figures and Tables

**Table 1:** RT–qPCR primers.

Gene	Sequence
*SMAD6*–F	GTGAATTCTCAGACGCCAGC
*SMAD6*–R	CTGCCCTGAGGTAGGTCGTA
*AK4*–F	CCCTCCTAGCGGAAGGGTAT
*AK4*–R	GCACTCCTCGGCTCTTGTA
*METRNL*–F	GCTGGTTAGGAGGCACAGG
*METRNL*–R	AGCCTCGAACGGCGAAG
*mt–ND4*–F	AAGTCAAAAAGCTATTA
*mt–ND4*–R	CTTACATCCTCATTACTATTC
*mt–ND4L*–F	CTCACACCTCATATCCTCCCTAC
*mt–ND4L*–R	GCTAAGAGGGAGTGGGTGTT
*mt–ND5*–F	ACCGCTAACAACCTATTCCAACTG
*mt–ND5*–R	GATTGCTTGAATGGCTGCTGTG
*β–Actin*–F	TCTTCCAGCCTTCCTTCCTG
*β–Actin*–R	CAATGCCAGGGTACATGGTG

## Data Availability

All data are available upon request. Transcriptomic data has been submitted to the Sequence Read Archive of the National Center for Biotechnology Information, the accession number for which is PRJNA1128853.

## Supplementary Materials

The following supporting information can be downloaded at https://www.mdpi.com/article/10.3390/metabo14070369/s1, Figure S1: HPLC chromatograms of ATP in SH-SY5Y cells; Figure S2: Flow cytometry graphs of ROS in SH-SY5Y cells.
