# Supplementary material for: Impaired Mitochondrial Energy Metabolism Regulated by p70S6K: A Putative Pathological Feature in Alzheimer’s Disease
Source: Metabolites. 2024 Jun 29;14(7):369. doi: 10.3390/metabo14070369 (PMC11278668; doi:10.3390/metabo14070369)
Supplement: Supplementary file 1 [file metabolites-14-00369-s001.zip › metabolites-3055920-supplementary.pdf]

Supplementary Materials

Impaired Mitochondrial Energy Metabolism Regulated by *p70S6K*: A Putative Pathological Feature in Alzheimer's Disease

Wenyu Gu [1,2,3], Xinli Cong [1,2,3], Yechun Pei [1,2,3], Nuela Manka'a Che Ajuyo [1,2], Yi Min [1,2,3,*] and Dayong Wang [1,2,*]

[1] Key Laboratory of Tropical Bioresources of the Educational Ministry of China, School of Pharmaceutical Sciences, Hainan University, Haikou 570228, China

[2] Laboratory of Biopharmaceuticals and Molecular Pharmacology, One Health Cooperative Innovation Center, Hainan University, Haikou 570228, China

[3] Department of Biotechnology, School of Life and Health Sciences, Hainan University, Haikou 570228, China

* Correspondence: 992601@hainanu.edu.cn (Y.M.); wangdy@hainanu.edu.cn (D.W.); Tel.: +86-136-3761-6384 (Y.M.); +86-187-8955-6728 (D.W.)

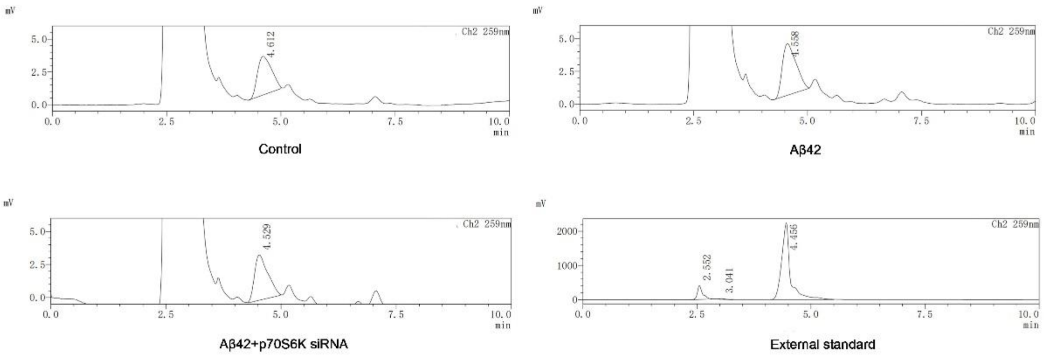

Figure S1. HPLC chromatograms of ATP in SH-SY5Y cells. The retention times of ATP peaks were indicated on the graphs. SH-SY5Y cells were treated with Aβ42 for 24 h with or without knockdown of p70S6K gene expression, n = 3.

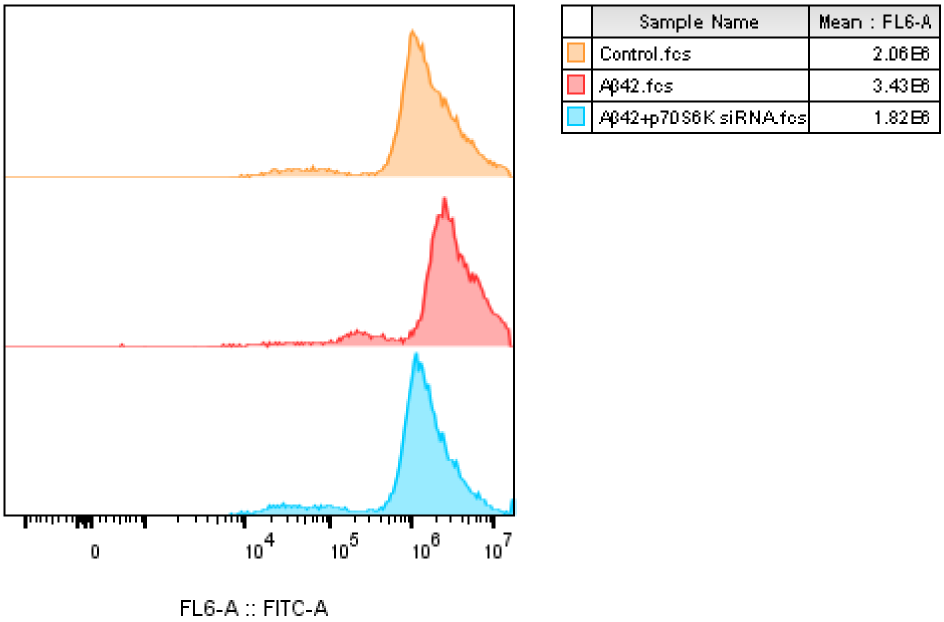

Figure S2. Flow cytometry graphs of ROS in SH-SY5Y cells. SH-SY5Y cells were treated with Aβ42 for 24 h with or without knockdown of p70S6K gene expression. The cellular fluorescence intensity in the FITC channel quantified to detect intracellular ROS levels. Yellow represents the control, red represents the Aβ42-treated group, blue represents the p70S6K siRNA- and Aβ42-treated group. The mean values in the table represent the levels of ROS, n = 3.
